# Simultaneous visualization of DNA loci in single cells by combinatorial multi-color iFISH

**DOI:** 10.1038/s41597-022-01139-2

**Published:** 2022-02-10

**Authors:** Ana Mota, Maud Schweitzer, Erik Wernersson, Nicola Crosetto, Magda Bienko

**Affiliations:** 1 Bienko-Crosetto Lab for Quantitative Genome Biology, Department of Microbiology, Tumor and Cell Biology, Karolinska Institutet, Stockholm, SE-17165 Sweden; 2 Science for Life Laboratory, Tomtebodavägen 23 A, Solna, SE-17165 Sweden

**Keywords:** Fluorescence in situ hybridization, Oligonucleotide probes, Wide-field fluorescence microscopy

## Abstract

Single-molecule DNA fluorescence *in situ* hybridization (FISH) techniques enable studying the three-dimensional (3D) organization of the genome at the single cell level. However, there is a major unmet need for open access, high quality, curated and reproducible DNA FISH datasets. Here, we describe a dataset obtained by applying our recently developed iFISH method to simultaneously visualize 16 small (size range: 62–73 kilobases, kb) DNA loci evenly spaced on chromosome 2 in human cells, in a single round of hybridization. We show how combinatorial color coding can be used to precisely localize multiple loci in 3D within single cells, and how inter-locus distances scale inversely with chromosome contact frequencies determined by high-throughput chromosome conformation capture (Hi-C). We provide raw images and 3D coordinates for nearly 10,000 FISH dots. Our dataset provides a free resource that can facilitate studies of 3D genome organization in single cells and can be used to develop automatic FISH analysis algorithms.

## Abstract (summary)

**Table:** 

Measurement(s)	DNA loci 3D coordinates
Technology Type(s)	Fluorescence *In Situ* Hybridization
Factor Type(s)	DNA FISH probe target (locus)
Sample Characteristic - Organism	Homo sapiens

Machine-accessible metadata file describing the reported data: 10.6084/m9.figshare.17281358

## Background & Summary

In eukaryotic cells, the genome is highly structured and is characterized by distinct chromatin arrangements at different length scales^1,2^. The three-dimensional (3D) organization of the genome influences and is influenced by fundamental nuclear processes, including DNA replication, transcription and DNA repair^3–7^. Numerous studies based on high-resolution DNA fluorescence *in situ* hybridization (FISH) have highlighted how the 3D genome is highly heterogeneous across different cells of the same type and genetic background, although it is structured following the same folding principles^8,9^. For example, whereas in most cell types chromosomes are organized in chromosomal territories^10^—clearly separated spatial units that tend to occupy non-random radial locations in the nucleus—there is a high degree of cell-to-cell variability in the exact placement of individual chromosomes, including homologues^11^. Similarly, visualization of topologically associating domains (TADs)^12^ by high-throughput sequential FISH techniques has revealed a great amount of variability in the compaction and relative positioning of these domains between different cells of the same type^13–19^. Although very powerful, sequential FISH techniques are prone to the accumulation of mechanical shifts during multiple iterations of hybridization and imaging, resulting in progressive drift of the coordinates of the FISH signals (fluorescent dots) and potentially increasing background noise and causing artefacts. A complementary approach—although intrinsically endowed with lower throughput—is based on spectral barcoding of multiple DNA FISH probes, where the information about a given locus is encoded in a color combination by labelling a locus of interest with multiple fluorescent dyes simultaneously^20^. In principle, an approach combining spectral barcoding with sequential hybridization and imaging could significantly reduce the number of hybridization cycles currently used in high-throughput DNA FISH approaches.

**Fig. 1:**
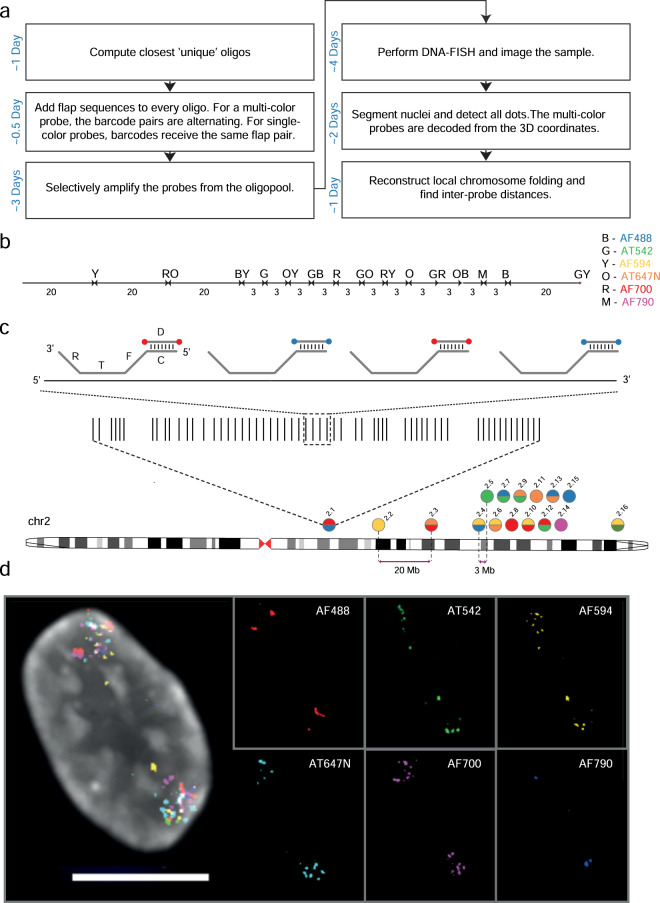
Overview of miFISH and experimental design used to obtain Dataset 1^23^ and 2^24^. (**a**) miFISH workflow from probe generation to analysis output. (**b**) Inter-probe distance between all the 16 miFISH probes along chr2. Letters indicate the fluorescent dye(s) used to label each probe, as indicated on the right. (**c**) Genomic location of the 16 miFISH probes along chr2 and of the oligos (vertical black bars) composing probe 2.1 as an example. Each probe is displayed as a filled circle either in one (single-color probes) or two (dual-color probes) colors, depending on the combination of fluorescently labeled detection (D) oligos used. In dual-color probes, the oligos are labeled with fluorophores (filled colored circles) in alternating colors, as shown for four consecutive oligos encircled by the dashed square, as an example. Each probe consists of 700 oligos with the structure shown on the top, and the consecutive probes are spaced either 3 Mb or 20 Mb apart as shown below chr2 ideogram and in (**c**). T, target sequence. F and R, adaptor sequences used to selectively amplify all the oligos composing a probe from a complex oligopool containing 12,000 oligos (see Methods). C, color sequence to which a complementary detection (D) oligo binds. See Supplementary Table 1 for the list of all oligos in each of the 16 probes. (**c**) Microscopy image showing one example of RPE cell nucleus in which we detected all the 16 miFISH probes displayed in (**b**) and (**c**). Scale bar: 10 µm. The smaller panels correspond to the 6 fluorescent dyes shown in (**b**) imaged separately. The images were deconvolved using the Huygens software, as described in the Methods. Grey, DNA stained with Hoechst 33342.

**Fig. 2:**
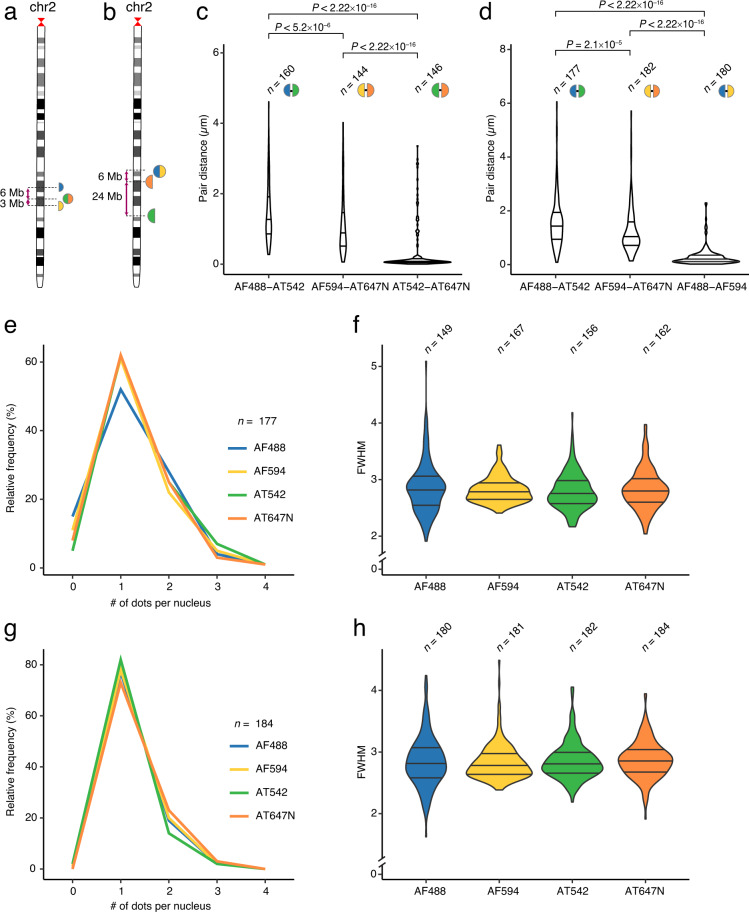
Co-localization analysis for dual-color miFISH probes. (**a**,**b**) Chr2 ideograms showing the location of two different sets of three miFISH probes (colored circles) including one dual-color probe and two single-color probes targeting DNA loci 3 and 6 Mb apart (**a**) or 6 and 24 Mb apart (**b**) (Datasets 3^25^ and 4^26^). Probe colors are the same as in Fig. 1c. (**c**) Distributions of pairwise distances between DNA FISH dots corresponding to different pairs of colors for the probes shown in (**a**). *n*, number of clusters in which the corresponding color pair was detected. *P*, p-value (Wilcoxon test, two-tailed). (**d**) Same as in (**c**), but for the probes shown in (**b**). (**e**) Distributions of the number of dots per nucleus in each channel, for the probes shown in (**a**). (**f**) Distributions of full width half maximum (FWHM) values in each channel, for the probes shown in (**a**). *n*, number of dots. The channels are ordered from left to right by increasing wavelength. (**g**) Same as in (**e**), but for the probes shown in (**b**). (**h**) Same as in (**f**), but for the probes shown in (**b**). In all the violin plots in the figure, the horizontal lines indicate the first, second (median) and third quartile (from bottom to top), and the whiskers extend from the minimum to the maximum value.

**Fig. 3:**
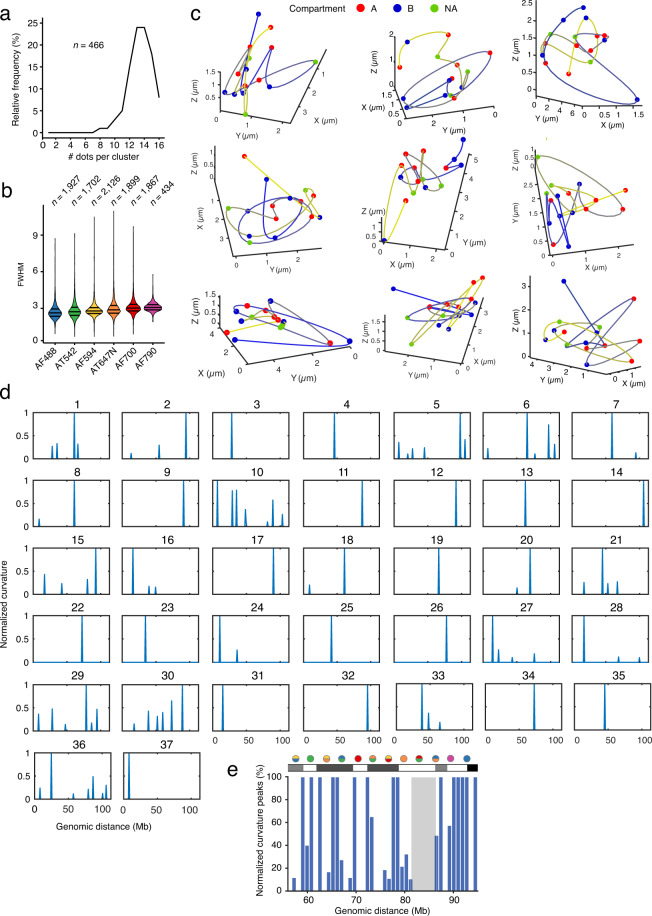
Visualization of chromosome structures. (**a**) Frequency distribution of the number of dots (probes) detected in 466 (*n*) miFISH dot clusters in G1 cells (Dataset 1^23^ and Methods). (**b**) Distributions of the full width half maximum (FWHM) values of the dots identified in 6 different channels for the miFISH chr2 Replicate 1 experiment (Dataset 1^23^). *n*, number of dots. The channels are ordered from left to right by increasing wavelength. In all the violin plots, the horizontal lines indicate the first, second (median) and third quartile (from bottom to top), and the whiskers extend from the minimum to the maximum value. (**c**) Examples of interpolated structures for 9 out of 466 miFISH dot clusters summarized in (**a**). Colored dots represent individual probes whereas the connecting lines were drawn by interpolation using the cubic splines curve method (see Methods). Color gradient, from yellow to blue, follows the genomic path of the DNA segment probed (see Fig. 1c). Each dot is colored based on the A/B compartment assignment of the corresponding probe. (**d**) Normalized curvature peaks in 37 interpolated structures such as those shown in (**a**), in which all the 16 miFISH probes were identified. (**e**) Normalized curvature peaks pooled from all 37 structures shown in (**d**) for the genomic coordinates 55–95 Mb where equal inter-probe spacing (every 3 Mb) was applied. Probes are colored following the scheme shown in Fig. 1c. The Cytobands corresponding to this segment of chr2 are shown above the plot. The grey shading depicts a region depleted of curvatures.

**Fig. 4:**
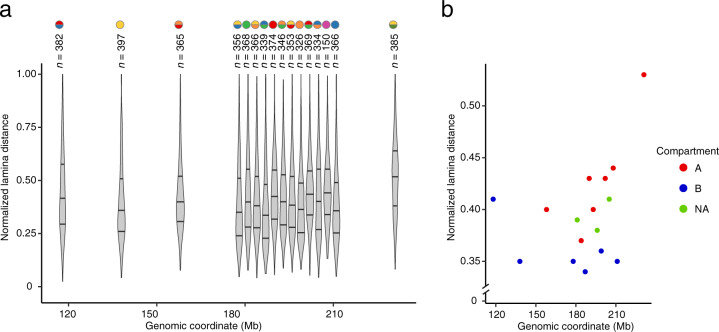
Radial localization of miFISH probe targets. (**a**) Distributions of the normalized 3D distances to the nuclear lamina of all the dots identified using the 16 miFISH probes (Dataset 1^23^) (see Methods for how the normalization was done). Each colored circle on top of the violin plots represents one probe. Colors are the same as in Fig. 1c. In all the violin plots, the horizontal lines indicate the first, second (median) and third quartile (from bottom to top), and the whiskers extend from the minimum to the maximum value. (**b**) Median normalized lamina distances by genomic coordinate for the 16 probes shown in (**a**). Each dot represents one of the 16 miFISH probes and is colored based on the A/B compartment assignment of the corresponding genomic target.

**Fig. 5:**
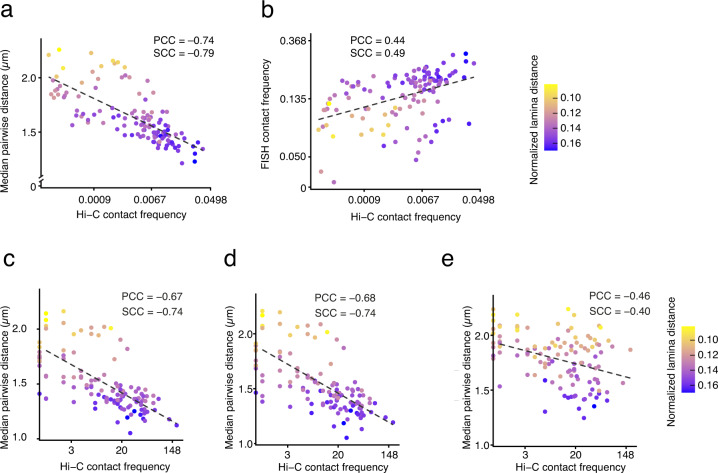
Comparison with Hi-C. (**a**) Correlation between the median pairwise distances and the corresponding contact frequencies determined by Hi-C for all the 16 miFISH probes (Dataset 1^23^). *n*, number of probe pairs. (**b**) Correlation between the contact frequencies between each pair of 16 miFISH probes (whereby dots located 1 μm or less apart are considered as being in contact) and the corresponding contact frequencies measured by Hi-C. (**c**) Same as in (**a**) but using less stringent dot assignment criteria by allowing the possibility that dots are assigned to multiple dual-color probes. (**d**) Same as in (**a**) but using a more relaxed threshold (0.55 µm) for the overlap between two dots. (**e**) Same as in (**a**) but using a different pipeline that allocates dots in a random manner to a probe. In all the plots in the figure, the dashed lines represent linear regression. PCC, Pearson’s correlation coefficient. SCC, Spearman’s correlation coefficient. Each dot is colored based on the normalized distance from the lamina (see Methods for how the normalization was done). In all the scatter plots in the figure, each dot represents one of 120 possible probe pairs using the 16 miFISH probes.

**Fig. 6:**
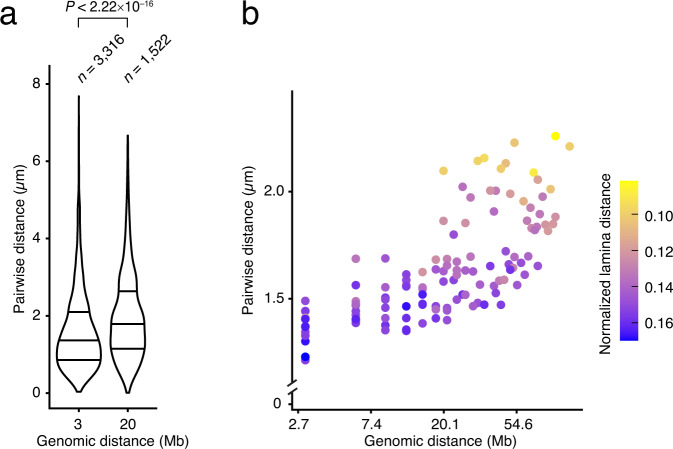
Correlation between genomic and physical distances. (**a**) Distributions of all possible pairwise distances, separately for the subset of 16 miFISH probes spaced 3 or 20 Mb apart on the linear genome. *n*, number of probe pairs analyzed. *P*, p-value (Wilcoxon test, two-tailed). In the violin plots, the horizontal lines indicate the first, second (median) and third quartile (from bottom to top), and the whiskers extend from the minimum to the maximum value. (**b**) Correlation between the median pairwise 3D distances and the corresponding genomic distances for all possible pairs of 16 miFISH probes (Dataset 1^23^). The pairwise distances are displayed in logarithmic scale on both axes. Each dot represents one of 120 possible probe pairs using the 16 miFISH probes. *n*, number of probe pairs analyzed. From 20 Mb upwards, the pairwise distances increase exponentially. Each dot is colored based on the normalized distance from the lamina (see Methods for how the normalization was done).

**Fig. 7:**
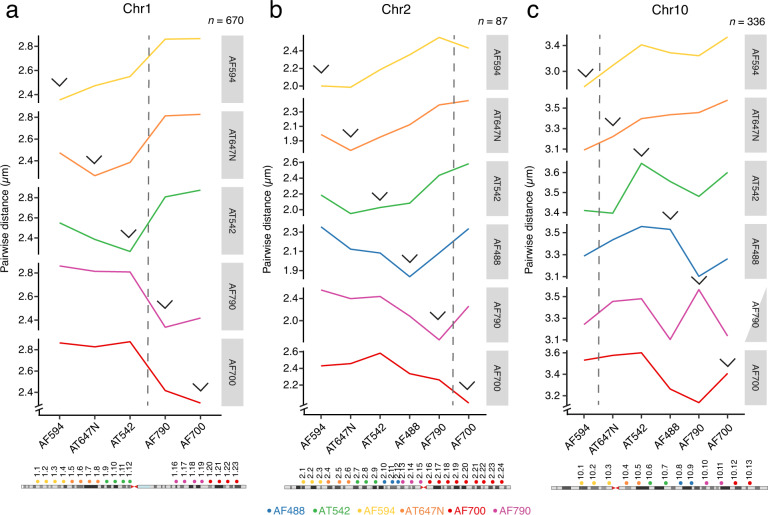
Chromosome spotting using 57 single-color iFISH probes. (**a**–**c**) The location of the probes is shown above each chromosome ideogram on the bottom. The plots represent the 3 different iFISH experiments involving the probes for chr1 in (**a**), chr2 in (**b**) and chr10 in (**c**). The line plots connect the median pairwise distances of a probe group that shares the same color against another probes group. Each line uses a group of probes as a reference, which are mentioned on the right of each line plot, to calculate pairwise distance against the other color groups including the probes group that share the same color (pointed by the blue arrows). The pairwise distances against the same probe are excluded. The grey dashed line shows centromere location. See Supplementary Table 2 for the list of oligos in each probe. *n*, number of iFISH dot clusters in G1 cells (Dataset 5^28^, 6^29^ and 8^31^, and Methods).

Despite the increasing number of applications of high-resolution DNA FISH techniques in the field of 3D genome organization^21^, there is a lack of high quality, open-access, and curated DNA FISH datasets. These could be used, for example, to develop and train machine learning algorithms for automatically detecting DNA FISH signals or to test theoretical models of 3D genome organization. More simply, such data, together with the accompanying analytical tools made publicly available, would represent immensely valuable resources for researchers who are new to DNA FISH and need to familiarize themselves with the intricacies of the data generated by this technique. To fill this gap in, here we describe a multi-color DNA FISH (miFISH) approach based on our recently developed iFISH method^22^, which allows for simultaneous visualization of multiple DNA loci in single cells, using combinatorial color indexing (spectral barcoding) of individual loci. We provide two replicate miFISH datasets (Datasets 1 and 2^23,24^) obtained by using 10 combinatorially labelled probes and 6 single-color probes targeting 16 short-sized (mean size: ~66 kilobases, kb) loci on human chromosome (chr) 2, where consecutive targets are separated by either 3 or 20 megabases (Mb) to generate a large spectrum of inter-probe distances along the chr2 q-arm (Fig. 1 and Table 1). Additionally, we provide two datasets (Datasets 3 and 4^25,26^) containing a single dual-color probe alongside singly labelled probes, used to identify thresholds for calling co-localization events (Fig. 2 and Table 1). Using these data, we identify 740 clusters of signals corresponding to individual alleles of chr2, which we use to study single chromosome topologies, highlighting extreme cell-to-cell variability in how DNA folds at sub-chromosomal resolution in interphase nuclei (Fig. 3). We validate miFISH by quantifying the distance to the nuclear lamina of FISH dots located in different chromosome compartments (Fig. 4), by comparing pairwise dot distances with chromosome contact frequencies measured by high-throughput chromosome conformation capture (Hi-C)^27^ (Fig. 5), and by assessing how pairwise dot distances scale with genomic distance (Fig. 6). Finally, we provide five additional datasets (Datasets 5–9^28–32^) obtained using only single-color probes targeting multiple loci evenly spaced every 10 Mb along chr1, 2 and 10, to test whether the observations based on miFISH data can be recapitulated using single-color probes (Fig. 7 and Table 1). For every dataset, we provide raw images, 3D FISH dot coordinates after shift and chromatic aberration correction, and all the scripts needed to reproduce the analyses presented here.

**Table 1:** List of datasets provided.

Dataset #	Ref.	Description	Chr	# Probes	# FOVs	Size (GB)
1	^23^	miFISH with 16 multi-color probes (Replicate 1)	2	16	25	26
2	^24^	miFISH with 16 multi-color probes (Replicate 2)	2	16	19	23
3	^25^	miFISH with one dual-color probe (AF594-AF488) and two single-color probes (AT546 and AT647N)	2	3	7	4
4	^26^	miFISH with one dual-color probe (AT647N-AT542) and two single-color probes (AF594 and AF488)	2	3	10	5
5	^28^	iFISH chr1 spotting (Replicate 1)	1	23	50	46
6	^29^	iFISH chr2 spotting (Replicate 1)	2	24	7	8
7	^30^	iFISH chr2 spotting (Replicate 2)	2	24	50	53
8	^31^	iFISH chr10 spotting (Replicate 1)	10	13	30	23
9	^32^	iFISH chr10 spotting (Replicate 2)	10	13	10	12

## Methods

### miFISH probe design

A scheme of miFISH and of the workflow that we used to generate the datasets described here is shown in Fig. 1a. We designed 16 miFISH probes targeting 16 loci on chr2 using the iFISH4U webtool (www.ifish4u.org), which we previously described^22^. The probes are distributed along the q-arm of chr2, and consecutive probes are spaced either 3 or 20 Mb apart (Fig. 1b). The probe span (*i.e*., the genomic distance from the coordinate of the first base in the 5′ oligonucleotide (oligo) to the last base of the 3′ oligo in the probe) is 66 ± 2.7 kb (mean ± s.d.). The sequences of all the oligos composing each probe are available in the Supplementary Table 1. Each probe consists of 700 oligos, and each oligo is 100 nucleotides (nt) long. The oligo sequence contains two 20 nt adapter sequences (F and R) for PCR amplification, a 40 nt sequence complementary to the DNA target (T), and a 20 nt color barcode (C) to which a detection fluorescently labelled oligo can be hybridized (Fig. 1c). We purchased all the oligos as a single 12k oligopool (Twist Biosciences) and produced the 16 probes individually using the iFISH pipeline^22^. To index each probe and make it distinguishable from all the others, we designed the F and R adapter sequences of the oligos such that 10 of the probes could be labelled with two alternating colors (out of 6 colors that can be resolved using our microscope), while the remaining 6 probes were labelled with a single color (Fig. 1c). Additionally, we retrieved 57 probes targeting multiple loci evenly spaced along chr1, 2 and 10, each labeled with a single color, from the iFISH probe repository that we previously established^22^. The sequences of all the oligos composing these probes are available in the Supplementary Table 2.

### miFISH probe production

To produce the FISH probes, we amplified the oligos corresponding to each probe by real-time PCR using the SYBR Select Master Mix (Thermo Fisher Scientific) and a probe-specific combination of primers complementary to the F and R adapter sequences (see Fig. 1c). We purchased all the primers from Integrated DNA Technologies (IDT) as standard desalted oligos. We purified the PCR products with Agencourt AMPure XP beads (Beckman Coulter) and measured the DNA concentration using the Qubit dsDNA HS Assay Kit (Thermo Fisher Scientific). Next, we converted each individual PCR product (probe) into RNA using the HiScribe T7 Quick High Yield RNA synthesis kit (New England Biolabs). We carried out the *in vitro* transcription (IVT) reaction at 37 °C for 16 h in a final volume of 30 μL containing 1 μg of purified PCR product, 6.67 mM of dNTPs (Thermo Fisher Scientific), 2 units of RNaseOUT Recombinant Ribonuclease Inhibitor (Thermo Fisher Scientific) and 2 μL of T7 RNA polymerase mix. We purified the amplified RNA with Agencourt RNAClean XP beads (Beckman Coulter) and measured the concentration with the Qubit RNA BR assay (Thermo Fisher Scientific). We then converted the purified RNA into cDNA by reverse transcription (RT) using the Maxima H Minus Reverse Transcriptase (Thermo Fisher Scientific) and a primer carrying the C adapter sequence serving as docking site for the fluorescently labeled detection oligo. We carried out the RT reaction at 50 °C for 1 h, in a volume of 20 μL containing 15 μg of purified RNA, 1.5 mM of dNTPs, 20 μM of the corresponding primer, 1x reverse transcription buffer, 10 units of Maxima H Reverse Transcriptase and 2 units of RNaseOUT (Thermo Fisher Scientific). We then incubated the reaction at 85 °C for 5 min to inactivate the enzymes. To remove the template RNA, we added 20 μL of 0.5 M EDTA (Thermo Fisher Scientific) and 20 μL of 1 M NaOH (Sigma Aldrich) directly into the RT reaction, incubated the samples at 95 °C for 15 min and immediately purified the single-stranded DNA (ssDNA) using Oligo Binding Buffer (Zymo Research) and Zymo-Spin IC columns (Zymo Research). We eluted each probe in 40 μL Nuclease-Free Water (Thermo Fisher Scientific) and measured the DNA concentration with the Qubit ssDNA Assay Kit (Thermo Fisher Scientific). We stored the probes at −20 °C prior to DNA FISH experiments.

### Sample preparation

We performed all the experiments in hTERT RPE-1 cells (American Tissue Culture Collection). We grew the cells in Dulbecco’s Modified Eagle’s Medium/Nutrient Mixture F-12 (DMEM: F12, ATCC) supplemented with 10% FBS (Sigma). This cell line is not included in the ICLAC database of commonly misidentified cell lines. We regularly checked the cells for Mycoplasma contamination but did not authenticate them. We incubated the cells at 37 °C in 5% O_2_ and 5% CO_2_. When the cells reached 80% confluency on coverslips (thickness 1.5 µm, 18 × 18 mm, VWR), we processed them following an adapted version of the 3D-FISH protocol described before^33^. Unless otherwise specified, we performed all the incubations at room temperature using solutions either stored or pre-equilibrated at the same temperature. Briefly, we fixed the cells in 1x PBS (Thermo Fisher Scientific)/4% formaldehyde (EMS) for 10 min, followed by quenching of the unreacted formaldehyde in 1x PBS/125 mM glycine (Sigma Aldrich) for 5 min. Subsequently, we washed the coverslips three times, 5 min each with 1x PBS (Thermo Fisher Scientific)/0.05% Triton X-100 (Promega) and permeabilized the cells in 1x PBS/0.5% Triton X-100 for 20 min, then washed twice for 5 min with 1x PBS/0.05% Triton X-100. Afterwards, we incubated the coverslips in 0.1 N HCl for 5 min and quickly rinsed them twice in 1x PBS/0.05% Triton X-100. Lastly, we rinsed the coverslips in 2x SSC buffer (Thermo Fisher Scientific) and incubated them overnight in 2x SSC/50% formamide (Thermo Fisher Scientific)/50 mM sodium phosphate (Sigma Aldrich). The following day, we transferred the coverslips to +4 °C and kept them for one week in 2x SSC/50% formamide/50 mM sodium phosphate. Lastly, we exchanged the buffer to 2x SSC at +4 °C and stored the samples in this buffer for up to 1 month.

### miFISH probe hybridization based on 3D DNA FISH

We performed DNA FISH following an adapted version of the 3D-FISH protocol previously described^33^. Unless otherwise specified, we performed all the incubations at room temperature using solutions either stored or pre-equilibrated at the same temperature. We first immersed the coverslips in a pre-hybridization buffer (PHB) containing 2x SSC/5x Denhardt’s solution (Thermo Fisher Scientific)/50 mM sodium phosphate buffer/1 mM EDTA/100 ng/μL ssDNA (Invitrogen)/50% formamide, pH 7.5–8.0, and incubated them for 1 h at 37 °C in a humidity chamber. During this time, we prepared the first hybridization mix (HM-1) by mixing each probe at 1:9 vol/vol ratio with 1.1x first hybridization buffer (HB-1) containing 2.2x SSC/5.5x Denhardt’s solution/55 mM sodium phosphate buffer/1.1 mM EDTA/111 ng/μL ssDNA/55% formamide/11% dextran sulfate (Sigma Aldrich) pH 7.5–8.0 (in this mix, the final concentration of each oligo is 0.05 nM). We then removed the coverslips from PHB and placed it on top of 20 μL of HM-1 deposited onto a microscope slide. We sealed the coverslips with fixogum (MP Biomedical) and waited until the fixogum solidified. Next, we performed DNA denaturation by placing the coverslips for 2 min 30 s at 75 °C on a heating block and incubated the samples for 15–18 h at 37 °C. The next day, we washed the coverslips twice, 5 min each at 65 °C in 0.2x SSC/0.2% Tween (Promega) pre-warmed at 65 °C inside a water bath, followed by a brief wash in 4x SSC/0.2% Tween at room temperature, a rinse in 2x SSC, and then exchanged the solution with 2x SSC/25% formamide. Next, we immersed the coverslips in 200 μL of the second hybridization mix (HM-2) containing the six secondary fluorescently labelled oligonucleotides (one per color), each at a final concentration of 20 nM in HB-2 containing 2xSSC/25% formamide/10% dextran sulfate/1 mg/mL *E.coli* tRNA (Sigma Aldrich)/0.02% bovine serum albumin (Thermo Fisher Scientific), and incubated them for 24 h at 30 °C. Afterwards, we washed the coverslips for 1 h at 30 °C in 2x SSC/25% formamide, followed by 30 min at 30 °C in 1.23 ng/mL Hoechst 33342 (Sigma Aldrich) in 2x SSC/25% formamide. Lastly, we briefly rinsed the coverslips twice in 2x SSC, before mounting them with ProLong Diamond Antifade Mountant (Thermo Fisher Scientific).

### Image acquisition

We imaged all the samples using a CFI Plan Apochromat Lambda 100X Oil objective (NA 1.45, WD 0.13 mm) (Nikon) mounted on a custom-built Eclipse Ti-E inverted microscope system (Nikon) controlled by the NIS Elements software (Nikon) and equipped with an iXON Ultra 888 EMCCD camera (Andor Technology). For each sample, we acquired multiple image stacks, each consisting of 81–95 focal planes spaced 0.2 μm apart. All the optical components required to achieve a clear separation between the 7 channels are listed in Table 2.

**Table 2:** List of filters and dichroic mirrors for each dye used in miFISH.

Dye	Excitation filter	Dichroic mirror	Emission filter
Hoechst 33342	390/22 (Lumencor)	440/40 (custom-made Polychroic 1 from Chroma)	FF01-447/60–25 (Semrock)
AF488	FF01-494/20–25 (Semrock)	FF506-Di 03-25x36 (Semrock)	FF01-527/20–25 (Semrock)
AT542	FF01-534/20-25 (Semrock)	570/20 (custom-made Polychroic 1 from Chroma)	FF01-567/15-25 (Semrock)
AF594	FF01-586/20-25x5 (Semrock)	630/27 (custom-made Polychroic 2 from Chroma)	FF01-628/32-25 (Semrock)
AT647N	631/28 (Lumencor)	675/29 (custom-made Polychroic 1 from Chroma)	FF01-676/29-25 (Semrock)
AF700	ZET690/10x (Chroma)	622/35 (custom-made Polychroic 2 from Chroma)	ET740/40x (Chroma)
AF790	780 nm MaxLine laser clean-up filter (Semrock)	808/35 (custom-made Polychroic 1 from Chroma)	FF01-832/37-25 (Semrock)

### Correction of shifts and chromatic aberrations

To correct for chromatic aberrations and shifts between the channels, we imaged TetraSpeck Microspheres (0.1 μm, fluorescent blue/green/orange and dark red, Thermo Fisher Scientific) before each imaging session. We used the DNA stain channel as the reference channel and determined the location of the beads by fitting a 2D Gaussian profile in *x, y* and a 1D Gaussian in *z*. To this end, we implemented a previously described algorithm^34^ for measurement and correction of chromatic aberrations in MATLAB (https://github.com/elgw/df_cc). The algorithm uses a second-order polynomial over the lateral plane (*x*, *y*) and a constant shift in the axial (*z*) direction. We used the channel of the ATTO 647 N (AT647N) dye as reference, *i.e*., we transformed the images from the other channels to match AT647N. The mean square errors (MSE) of the distances between individual pairs of points before and after the correction are listed in Table 3. Although this approach does not separate fitting errors from those caused by higher-order chromatic aberrations, these values can be used to set a conservative limit on the precision of our data points. Assuming errors to occur only in the axial direction, our measurements correspond to an MSE error for measuring pairwise distances of 24 nm at most.

**Table 3:** Mean square errors (MSE) calculated during the procedure for shift and chromatic aberration correction.

Dye	3D MSE before correction	2D MSE after correction	3D MSE after correction
AF488	1.62	0.07	0.11
AT542	0.69	0.04	0.12
AF594	6.79	0.09	0.11
AF700	0.65	0.06	0.12

AT647N was used as reference. All values are in pixels.

### Deconvolution and nuclei segmentation

After chromatic aberration correction, we performed automated 3D segmentation of cell nuclei stained with Hoechst 33342. To this end, we first deconvolved the DNA staining channel using the Huygens Professional Software (Scientific Volume Imaging, v17.04) with the following parameters: CMLE algorithm, null background, signal-to-noise ratio equal to 7, and 50 iterations. After deconvolution, we performed 3D segmentation of the nuclei in each field of view, using the tiff_auto3dseg script in the pygpseq Python3 package, which we previously described^22^ (https://github.com/ggirelli/pygpseq/).

### FISH dots localization

To identify FISH dots, we used our in-house image analysis suite DOTTER (available at github.com/elgw/dotter) written in MATLAB (MATLAB and Statistics Toolbox Release R2020a) and C99 with GSL (https://www.gnu.org/software/gsl/) to perform the following consecutive steps:

1. We detected all local maxima in the images using 6-connectivity, *i.e*., we considered a pixel a local maximum if it was brighter than its face neighbors.
2. We applied a difference of Gaussians (DoG) filter to the images by setting the DoG filter ***σ*** equal to 1.72 times the expected ***σ*** of the diffraction-limited dots in each channel.
3. For each channel, we inspected multiple fields of view by eye and manually selected a threshold that seemed to best capture true dots. DOTTER allows thresholding the DoG value of each local maximum for each color and as well as to threshold the full width half maximum (FWHM) value for each dot. This semi-automatic method attempts to detect dots in the most unbiased manner.
4. We applied the selected threshold to all the fields of view.
5. To localize each dot in 3D,we used a Gaussian function and integrated over each pixel individually according to the following procedure:

- We first fitted dots laterally to give ($\widehat{x}$, $\widehat{y}$)
- We then interpolated the axial profile *P*_*z*_ centered at ($\widehat{x}$, $\widehat{y}$)
- Lastly, we fitted $\widehat{z}$, from *P*_*z*_

We optimized a Maximum Likelihood functional^35^ using the simplex method implemented by the MATLAB function fminsearch using the following optimization parameters: location, background level, number of photons, and sigma. For performance reasons, we assumed the noise to be Gaussian.

To assess the localization precision of the dot fitting procedure, we generated synthetic miFISH images containing dots with characteristics as similar as possible to real FISH dots. Specifically:

1. We set the background level to the median value of each image
2. We simulated the sensor as having Gaussian noise with ***σ*** equal to 5
3. We set the peak brightness to either the min, max or mean dot intensity
4. We added Poissonian noise.
5. We generated 1,000 patches for each case, setting the true location uniformly randomly within the central pixel.

The smallest root-mean-square deviation (RMSE) was 6 nm in the AF594 channel, while the largest RMSE was 24 nm in the AT647N channel (Supplementary Table 3). The average RMSE in all the channels was 11 nm. Of note, we were not able to fit all the dots, especially in crowded regions where the dot locations might not converge locally.

### Identification of G1 cells

We identified G1 cells based on the signal intensity of the DNA stain, Hoechst 33342, in the nucleus. Briefly, we integrated the fluorescence intensity over each segmented nucleus (defined as a 2D binary mask) and over the *z* axis and then picked a threshold by applying the MATLAB function otsuthresh to the resulting fluorescence distribution histogram to separate two peaks: one (on the left in the histogram) corresponding to cells in the G1 phase and one (on the right in the histogram) representing G2/M cells.

### Calculation of radial distances

To calculate the distance of each identified miFISH dot from the nuclear lamina, we passed the output of DOTTER to the gpseq_fromfish script (v7.0.2) in our pygpseq package (10.5281/zenodo.5575870). For each FISH dot, the script computes its distance, *d_E*, to the nuclear edge as well as its distance, *d_C*, to the nuclear center. Specifically, *d_E* is the distance to the nearest background voxel of the segmented DNA stain image, while *d_C* is the distance to the nearest voxel in the top percentile of the distribution of distances from the nuclear edge. The script uses an anisotropic 3D Euclidean transform on the nuclear mask by applying the ndimage.morphology.distance_transform_edt function in the SciPy library (v1.2.1). Finally, the script computes the relative distance of each FISH dot to the nuclear edge as *d_E* / (*d_E* + *d_C*).

### Identification of dot clusters

To identify clusters of miFISH dots corresponding to loci located on the same homologue chromosome, we applied k-means clustering (implemented in DOTTER) and analyzed each cluster separately. This approach aims at partitioning all the dots found in each nucleus of G1 cells into two clusters corresponding to the two homologue chromosomes. The dots are divided in two different clusters according to the nearest mean (cluster centroid), and the process is iterated until the mean positions with the smallest dot distances within the cluster are found. We set the cluster radius to 40 pixels to enclose most of the dots found in each nucleus and to reduce the number of outliers. We then extracted the *x, y, z* coordinates of each dot in a .csv file.

### Dot allocation to the corresponding miFISH probe

We restricted our analysis to G1 cells containing two clearly identifiable dot clusters, corresponding to two chromosome homologues, and computed the 3D coordinates of all the dots in each cluster. For each cluster in a G1 cell, we expected to detect 10 signals for each fluorescent dye used except for the Alexa Fluor 790 (AF790) channel, where we expected 2 dots, since this channel was not included in any dual-color probe (Fig. 1c). Hence, for all the channels except AF790, we extracted the coordinates of the 10 brightest dots in each cluster, whereas for AF790 we extracted the coordinates of the 2 brightest dots in each cluster. To match signals coming from the same dual-color probe, we computed all pairwise distances between all the signals for all the channels. We expected the distance between signals originating from the same probe to be close to zero. We did, however, anticipate a shift due to chromatic aberrations between signals that in theory should be perfectly aligned. To measure this technical error and identify a cut-off for co-localization, we performed two sets of experiments, in which we used only one dual-color probe alongside two singly labelled probes targeting different loci on chr2 (Datasets 3, 4^25,26^ and Fig. 2a,b). We tested the shift for two different dual-color probes, where one of them could be imaged using the same multi-band dichroic mirror, while the other required a change of the cube holding dichroic mirrors. The median distance between two signals originating from the same probe labelled with ATTO 542 (AT542) and AT647N—which were imaged using the same multi-band dichroic mirror—was lower than 0.25 µm (Fig. 2c). In contrast, the median distance between two signals coming from the same probe labelled with Alexa Fluor 488 (AF488) and Alexa Fluor 594 (AF549)—for which dichroic mirrors were placed in different cubes and hence more mechanical movement was needed to image both—increased to 0.55 µm (Fig. 2d). Therefore, in further analyses, we retained only signals with pairwise distance lower than 0.55 µm for experiments in which dichroic mirrors were placed in different cubes, while for experiments in which dichroic mirrors shared the same cube, we set the threshold to 0.25 µm.

Next, we examined dots with pairwise distances lower than the defined cut-off distance and counted how often a certain dual-color probe is encountered in the list. We prioritized dual-color probes that were counted only once in the list, so that the corresponding dots could not be assigned to other dual-color probes. Given that the signal originating from a single FISH probe might split into two or more dots, we also implemented a cut-off value to filter out nearby dots of the same color and clear them from the list of other dual-color probes. When there was more than one option to assign a dot to a dual-color probe, we selected the dot pair with the shortest pairwise distance. Once we collected all the possible matches, we filtered out dots positioned more than 5 µm away from all the other dots in the same cluster and considered them outliers due to their location away from the cluster. We considered the dots not assigned to dual-color probes as single-color probes and assigned them to the brightest dot of the cluster that had not yet been selected. As single-color probes had twice as many oligos labelled with the same dye, we expected them to show a brighter signal in comparison to dual-color probes. Hence, we used this criterium to further increase the confidence of signal picking for single-color probes. Altogether, this approach yielded the expected dot counts per nucleus, with signals following the expected FWHM distribution (Fig. 2e–h).

### Visualization and geometrical analysis of miFISH dot clouds

As discussed above, we considered in our analysis only G1 cells with two clearly distinct clusters of miFISH dots and excluded signals originating from dual-color probes, when the expected dot pair was further apart than 0.25 or 0.55 µm depending on the dichroic mirror setup. As a result of such stringent criteria, we found 37 out of 466 (8%) clusters contained all the 16 probes, whereas most of the clusters contained 13–14 probes (Fig. 3a). As a further metric for correct dot picking, we used the FWHM value of the dots, which is the width of the intensity profile of a signal at half of the maximum intensity. This metric is dependent on the objective and emission wavelength of the dyes used, as shown in Table 4. We observed a relatively narrow distribution of the FWHM for the dots picked, with values increasing for dyes emitting in the higher range of the light spectrum and small deviations between the expected and observed FWHM values (Fig. 3b and Table 4), which might be explained by some small detection bias coming from our microscope setup.

**Table 4:** Predicted and observed full width half maximum (FWHM) values for each of the fluorophores used in this study.

Dye	Emission λ (nm)	Predicted FWHM (nm)	Predicted FWHM (px)	Observed FWHM (px)
AF488	525	282	2,17	2,61
AT542	562	302	2,32	2,73
AF594	617	332	2,55	2,79
AT647N	664	357	2,75	2,88
AF700	723	389	2,99	3,03
AF790	814	438	3,37	3,07

To visualize and compare the spatial distribution of the FISH dot clouds in each cluster in which all the 16 probes were identified, we interpolated the dots by fitting a cubic spline curve through the dot *x, y, z* coordinates using the MATLAB cscvn command. We observed substantial cluster-to-cluster variability in the shape of the resulting curves (Fig. 3c). To search for potential repeating patterns in the splines, we calculated all the curvatures present in each cluster as following:

1 $$k=\frac{\sqrt{{\left(z{\prime\prime} y{\prime} -y{\prime\prime} z{\prime} \right)}^{2}+{\left(x{\prime\prime} z{\prime} -z{\prime\prime} x{\prime} \right)}^{2}+{\left(y{\prime\prime} x{\prime} -x{\prime\prime} y{\prime} \right)}^{2}}}{{\left({x}^{{\prime} 2}+{y}^{{\prime} 2}+{z}^{{\prime} 2}\right)}^{\frac{3}{2}}}$$

We then normalized the curvature values by identifying the maximum curvature value for each cluster and dividing each curvature value of a given spline by that maximum curvature value of the same spline, so that the curvature values would range from 0 to 1. We only considered the curvature peaks above 0.1 for the following analysis. All the splines contained a unique pattern of curvatures, further highlighting the variability in the topology of individual chromosomes in single cells (Fig. 3d). A pooled analysis of all the curvature values did, however, reveal some structure of the q-arm of chr2, with clusters of chromosomal regions containing a high density of curvature peaks (thus, more twisting) interspersed with regions with no peaks (Fig. 3e). We emphasize, however, that this approach is primarily intended for visualization purposes, as the probes used are too far apart to enable an accurate reconstruction of the topology adopted by the chromatin fiber.

## Data Records

All the Datasets are listed in Table 1 and have been deposited in Figshare^23–26,28–32^ and are also available on the Image Data Resource (IDR) website with accession code idr0123. Each dataset consists of multiple fields of view (FOVs) that were acquired with seven different dyes (AF488 labeled as ‘a488’; AT542 as ‘tmr’; AF594 as ‘a594’; AT647N as ‘Cy5’, AF700 as ‘a700’, AF790 as ‘ir800’; and Hoechst 33342 as ‘dapi’) and all the FOVs are provided as separate.tiff files (Table 1). Each FOV comprises 81–95 focal planes spaced 0.2 μm apart and has a size of 1024 × 1024 pixels. These datasets files have already been corrected for chromatic aberrations, so each dataset includes information on the *x*, *y*, and *z* displacements from the center of the beads at different channels, as well as the number of beads used to calculate the displacements.

## Technical Validation

### Distribution of miFISH dots with respect to the nuclear lamina

The nuclear lamina influences the global chromatin architecture by instructing the assembly of the so-called lamina-associated domains (LADs), which consist of chromatin in a rather condensed state in contact with the nuclear periphery^4^ and are enriched in genomic regions belonging to the B compartment identified based on the Hi-C method^27^. Consequently, we hypothesized that the signals originating from the miFISH probes that target genomic loci belonging to the B compartment would be closer to the lamina than those originating from probes targeting loci located in the A compartment, also identified based on Hi-C. To test this hypothesis, we used our miFISH measurements (Datasets 1 and 2^23,24^) and measured the radial distance to the nuclear lamina for all the 16 miFISH probes using the approach described in the Methods. The distributions of the normalized distances of each of the 16 miFISH probes from the nuclear lamina are shown in Fig. 4a. Next, we assigned each FISH dot to either A or B compartment. We defined A and B compartments by extracting eigenvectors from the Hi-C data through PCA clustering, as previously described^27^. We downloaded Hi-C data obtained from RPE cells from the NCBI archive (GEO accession: GSE71831_RPE1-WT) and processed them with Juicer (https://github.com/aidenlab/Juicebox) following the developers’ instructions. The resulting Hi-C contact frequencies at 100 kb resolution are listed together with the corresponding miFISH probe pairs in Supplementary Table 4. Chromatin inside the A compartment is generally more open and active and is decorated with epigenetic histone marks for active transcription, while the B compartment encompasses regions decorated with repressive marks^27^. The sign of the Hi-C eigenvector is arbitrarily defined and typically correlates with epigenetic markers associated with inactive and repressive histone marks, such as histone 3 tri-methylated on lysine 9 (H3K9me3) for the B compartment, and histone marks indicative of active transcription such as histone 3 tri-methylated on lysine 4 (H3K4me3) for the A compartment. We restricted the A/B compartment calling to regions with an absolute eigenvector value greater than 0.01 to discard uncertain regions. As expected, we found that the lamina distances determined by miFISH (using Datasets 1 and 2^23,24^) were typically higher for probes belonging to A compartments than for probes of the B compartments (Fig. 4b). These results demonstrate that miFISH can reliably probe the location of DNA loci with respect to the nuclear lamina and in different chromatin compartments.

### Comparison with Hi-C

To further validate our miFISH measurements, we compared 3D pairwise distances measured by miFISH with the contact frequencies of the same pairs of genomic regions derived from Hi-C maps. However, pairwise distances measured by miFISH include long distances (often > 1 μm) that cannot be captured by Hi-C. To account for this, we used two approaches to compare miFISH data with Hi-C maps. In the first approach, we computed the median distance for all the 120 miFISH probe pairs using all the clusters that we detected with the miFISH pipeline, and then calculated the Hi-C contact ratio for all the pairs. The median pairwise distances of all the probe pairs was strongly anti-correlated (Pearson’s correlation coefficient, PCC: –0.74) with the interaction frequencies measured by Hi-C, as expected (Fig. 5a). In the second approach, we defined a threshold of 1 µm for calling as a contact a pair-wise distance measured by miFISH. Using this approach, we also observed a correlation, though weaker (PCC: 0.44), between contact frequencies measured by miFISH and Hi-C (Fig. 5b).

Next, we explored how pairwise distances between miFISH dots scale with Hi-C contact frequencies after varying the parameters used to assign miFISH dots to single- and dual-color probes. We first relaxed the dot assignment criteria by allowing dots corresponding to dual-color probes to be assigned to more than one probe, which resulted in a lower correlation (PCC: –0.67) with Hi-C, as expected (Fig. 5c). We then checked whether relaxing the co-localization threshold (0.55 µm instead of 0.25 µm) for channels imaged through the same multi-band dichroic mirror would result in a lower correlation with Hi-C. Indeed, the correlation decreased (PCC: –0.68), although not drastically (Fig. 5d). Lastly, we tested the correlation with Hi-C after assigning the dots randomly to the probes. To this end, we modified the procedure used for assigning each dot to the corresponding probe (see Methods) in the following manner. For dots corresponding to dual-color probes in which the second color was farther than 0.55 µm, we randomly assigned again the first dot until we found one pair of dots spaced less than 0.55 µm. For each cluster, we reiterated the dot assignment process 20 times, and we randomly allocated the dots in each probe 100 times. For each probe, we scored the dots based on the following criteria, in decreasing order of importance: (1) dot intensity; (2) same dot already allocated to another probe; (3) overlap distance lower than 0.55 µm; (4) dot allocation failed; (5) dots in the probe from the same channel with pairwise distance lower than 0.25 µm. Using this procedure, we observed a considerable drop in the correlation between miFISH and Hi-C (PCC: –0.46, Fig. 5e), validating our standard dot allocation procedure described in the Methods and thresholds (0.25 µm for channels imaged through the same multi-band dichroic mirror and 0.55 µm for the other channels) for assigning dots to the same dual-color probe. We therefore recommend using this approach to re-analyze our datasets or for the analysis of newly generated miFISH data.

### Relationship between pairwise distances and genomic distances

The higher-order structure of chromosomes remains understudied. Therefore, the possibility to target multiple genomic regions simultaneously using miFISH opens the opportunity to further unveil 3D chromosome organization. To this end, we compared pairwise distances between miFISH probes spaced 3 or 20 Mb apart (Fig. 1c). We expected to observe an increase in pairwise distances for probes separated by larger genomic distances. Indeed, the pairwise distances corresponding to the 6 miFISH probes placed 20 Mb apart were significantly higher than those of the 10 probes separated by 3 Mb (*P* = 2.2 × 10^–16^, Wilcoxon test, two-tailed) (Fig. 6a). We then examined the pairwise distances between all the probe pairs in all the clusters determined in G1 cells in our miFISH experiments (Datasets 1 and 2^23,24^). Again, we could observe a trend towards increased inter-probe pairwise distances for probes located at increasing genomic distances (Fig. 6b). Interestingly, the relationship between the genomic distance and pairwise distance for probes separated by less than 20 Mb did not follow an exponential increase as seen for probes separated by more than 20 Mb (Fig. 6b).

To further investigate the relationship between 3D and genomic distances, we performed additional iFISH experiments using a set of 57 singly labelled iFISH probes targeting multiple loci evenly spaced along chr1, 2 and 10, which we retrieved from our iFISH probe repository^22^ (Datasets 5–9^28–32^, Fig. 7a–c, and Supplementary Table 2). We calculated all the pairwise distances either between probes labelled with the same color or between probes labeled with two different colors and examined how the pairwise distances change with increasing genomic distances between the probes. In line with the above observations based on miFISH probes, we found that more distant genomic regions showed higher physical separation all along chr2 and the same was true for chr1, while in the case of chr10 such linear increase could not be observed along the entire chromosomal length, possibly indicating the existence of chromosome-specific higher-order organization patterns (Fig. 7a–c). Altogether, these results demonstrate that miFISH can be harnessed to study chromosome organization at different scales and test different models of chromatin folding.

## Usage Notes

### Considerations on how to minimize false positives in miFISH

iFISH probes typically consist of 96 oligos that need to bind cumulatively to the same genomic target to produce a detectable fluorescence signal. However, it is possible that individual oligos bind to multiple off-target genomic sites, thus increasing the fluorescence background and reducing the signal-to-noise. To minimize such possibility, we designed miFISH probes to contain a higher number (350) of oligos and increased the temperature in the washing step to 65 °C compared to 56 °C used in iFISH to prevent off-target binding upon hybridization. At the analytical level, we set stringent intensity thresholds aiming at capturing only the expected number of dots per nucleus in each channel, and then manually inspected each image to check if bright dots were not picked. One limitation of using miFISH probes containing many oligos is that the resulting FISH dots might ‘split’ into multiple dots corresponding to different parts of the targeted genomic region that fold together. This could result in the generation of false-positive dots that can bias downstream analyses. In general, we recommend filtering out dots with the same color that are closely located in 3D space (<0.50 µm apart). With respect to the procedure used to localize fluorescence dots in miFISH images, using only the pixel centers for dot localization would result in a mean absolute error of ~74 nm given the pixel size in our microscope setup (130 × 130 × 200 nm). Therefore, to achieve high localization precision in miFISH experiments, we performed Gaussian fitting as described above. We note that the fitting accuracy is predicted to scale with $\frac{\sigma }{\sqrt{N}}$ where *N* is the number of photons per dot and ***σ*** is the standard deviation of the Gaussian profile^36^. Therefore, the localization precision in miFISH (and, in general, in all single-molecule FISH assays) is strongly dependent on the quality of the images and on the levels of background fluorescence.

To maximize the accuracy of dot picking in miFISH, we relied on a supervised, semi-automated dot picking approach by manually curating all the dots automatically picked by DOTTER. Therefore, the miFISH datasets that we provide can now be used to train machine learning based unsupervised approaches that can then be applied to identify dots reliably and automatically in other high-resolution DNA FISH datasets. However, we emphasize that for any DNA FISH dataset to be suitable for training machine learning algorithms, the images must be of pristine quality, with as high as possible signal-to-noise.

## Supplementary information

Supplementary Table 1

Supplementary Table 2

Supplementary Table 3

Supplementary Table 4

## Data Availability

All the custom code written in MATLAB used for image processing and for all the analyses presented is available at https://github.com/anacmota/miFISH. The DOTTER suite written in MATLAB is available at github.com/elgw/dotter. The MATLAB code for chromatic aberration correction is available at https://github.com/elgw/df_cc. The Python documentation for 3D segmentation and lamina distance calculation is available here^37^.
